# Evaluation of an automated molecular diagnostic instrument for direct detection of Burkholderia pseudomallei from clinical specimens

**DOI:** 10.1099/jmm.0.002074

**Published:** 2025-09-22

**Authors:** Ian Gassiep, Matthew Glover, Mark Beecham, Brian Gorman, Melissa Page, James Stewart, Patrick N.A. Harris

**Affiliations:** 1 Faculty of Medicine, UQ Centre for Clinical Research, The University of Queensland, Royal Brisbane & Women’s Hospital, Queensland, Brisbane, Australia; 2 Department of Infectious Diseases, Mater Hospital Brisbane, Brisbane, Queensland, Australia; 3 Pathology Queensland, Royal Brisbane & Women’s Hospital, Queensland, Brisbane, Australia; 4 Pathology Queensland, Townsville University Hospital, Townsville, Queensland, Australia; 5 Pathology Queensland, Cairns Hospital, Cairns, Queensland, Australia; 6 Department of Infectious Diseases, Cairns Hospital, Cairns, Queensland, Australia

**Keywords:** *Burkholderia pseudomallei*, culture-independent, melioidosis, molecular detection, PCR, type III Secretion System (TTS1)

## Abstract

**Background.** Melioidosis is a potentially life-threatening infectious disease. The diagnosis of melioidosis is time-critical due to the organism’s intrinsic antimicrobial resistance and requirement for directed therapy.

**Aim.** To assess the ability of an automated molecular diagnostic instrument to detect *Burkholderia pseudomallei* directly from clinical samples.

**Methods.** Urine, sputum, swabs and Ashdown’s (ASH) broth were spiked with known concentrations of *B. pseudomallei* and analysed using an automated PCR platform (Panther^®^ Fusion; Hologic) targeting the type III Secretion System (TTS-1) gene. In addition, clinical specimens from patients with confirmed melioidosis were also evaluated.

**Results.** Urine was the clinical sample that demonstrated the lowest limit of detection (LOD), 1.8×10^2^ c.f.u. ml^−1^. Compared with dry swabs (LOD: 1.0×10^3^ c.f.u. ml^−1^), Amies agar swabs were inferior (LOD: >3.3×10^4^ c.f.u. ml^−1^). Inoculation of dry swabs into ASH, with an abbreviated incubation period, did not improve detection. All culture-positive sputum and urine samples from patients with confirmed melioidosis were detected by the PCR method.

**Conclusion.** This study demonstrates the ability of the Panther^®^ to directly detect *B. pseudomallei* across a range of clinical sample types and estimates the minimum bacterial concentration required for diagnostic detection. The described methodology holds promise for expediting diagnosis and, in turn, enhancing patient outcomes.

## Introduction

Time to diagnosis is a critical component in the management of sepsis. *Burkholderia pseudomallei* is the causative agent of melioidosis, an infectious syndrome that often results in bacteraemia. The case-fatality rate associated with this infection varies between regions, and in patients with septic shock, this rate can reach 90% [1].

A number of diagnostic challenges for melioidosis exist. First, culture is the mainstay of diagnosis. The inherent issue with culture as the primary mode of diagnosis is the time required. For bacteraemic melioidosis, the median time to positivity has been reported as 28–31 h [2,3]. Notably, this is only the time taken for the notification of a positive blood culture bottle, not the identification of the specific organism. Various diagnostic methods to identify *B. pseudomallei* directly from a positive blood culture sample have been reported [4,6]. Secondly, the laboratory identification of this organism is challenging, and misidentification is not uncommon [1].

Although bacteraemia is a common microbiological diagnosis of melioidosis, in some regions, 40% of culture-proven cases may be identified from alternate clinical samples, such as sputum, urine or pus [1]. Currently, there are limited methods for the rapid identification of *B. pseudomallei* from these samples.

The aim of this study was to create a method for identifying *B. pseudomallei* from various clinical samples using an automated molecular instrument, the Panther Fusion^®^ (Hologic, San Diego, CA, USA). The rationale for using this instrument is its current availability in Queensland, Australia, a melioidosis-endemic region, and its specificity of identification through molecular targets.

## Methods

The Panther^®^ is a fully automated PCR instrument that performs DNA extraction, purification and thermal cycling internally. The platform enables the utilization of Open Access functionality for conducting *in vitro* diagnostic assays. The TTS1-orf2 primers and probe were used for organism detection [7]. Based on previous data, the master mix consisted of 5 µl of 100 µM forward and reverse primers, 3.7 µl of 200 µM probe, 34.5 µl of potassium chloride, 4.25 µl of magnesium chloride and 8.5 µl of Tris buffer [8]. Additionally, 14 and 21 µl of a proprietary internal control primer and probe were included, respectively. The final master mix volume of 850 µl was created with purified molecular grade water, and Open Access RNA/DNA polymerase cartridges were included as per the manufacturer’s instructions. Thermocycler conditions included a 2 min hold stage at 95 °C and 45 cycles comprising 8 s at 95 °C and 25 s at 60 °C. A cycle threshold (Ct) of ≤43 was reported as detected, according to manufacturer settings. Each sample was run in duplicate and determined to be positive if at least one replicate was reported as detected.

A 0.5 McFarland suspension was created in sterile saline using a *B. pseudomallei* type strain, National Collection of Type Cultures (NCTC) 13178. From this standard, decreasing dilutions to a final concentration of 10^3^, 10^2^ and 10^1^ c.f.u. ml^−1^ were created. Aliquots of 20 and 50 µl from each dilution were inoculated in duplicate onto 5% horse blood agar (HBA) and incubated at 35 °C under aerobic conditions for 48 h to assess both purity and final concentration. Subsequently, these dilutions were used to create contrived samples. All isolate handling and sample preparation were conducted within a class II biosafety cabinet in a biosafety level 2 facility.

Sputum (*n*=24) and urine (*n*=25) samples were obtained from the Pathology Queensland Central laboratory. These samples were from hospitalized patients in a non-melioidosis-endemic region, without presumed or proven melioidosis, and had undergone standard laboratory analysis. For sputum, a 1 : 2 ratio of sputum to Sputasol^®^ (Thermo Fisher Scientific Inc.) was combined, vortexed for 10 s and incubated at 35 °C for 15 min. Subsequently, a 500 µl aliquot of this solution was transferred into a Hologic Specimen Aliquot Tube (SAT) containing 780 µl of lysis buffer.

For urine, a 500 µl sample was transferred from a sterile urine container into a SAT tube containing 600 µl of urine transport medium (Hologic).

To mimic sample collection from wounds, contrived samples were created using unused rayon swabs containing no media (dry swab) or Amies gel (Medical Wire and Equipment, Wiltshire, England). As pre-used clinical swabs have a wide variation in absorption, these were not assessed.

A 3 ml Ashdown’s (ASH) broth was spiked with 1 ml of dilution standard and incubated at 35 °C under aerobic conditions for 4 and 6 h. Following incubation, a 500 µl volume was transferred into SAT tubes for instrument analysis. Additionally, dry swabs were soaked in a known standard, swizzled into ASH broth, incubated for 4 and 6 h and subsequently tested as described above.

A centrifugation experiment was undertaken to determine the potential for concentration of organism DNA, and therefore improved detection. Samples were placed in a Hettich Rotina 380 centrifuge (Andreas Hettich GmbH and Co. KG, Germany) and spun at 3,000 r.p.m. for 5 min. A 500 µl sample was aliquoted from both the supernatant (top) and pellet (bottom), and analysed with the Panther^®^ as described above.

Finally, clinical samples from patients with proven melioidosis were examined to determine the real-world diagnostic utility of the assay. Patient samples were collected from Pathology Queensland Cairns and Townsville laboratories and were transported to the Central Laboratory in Brisbane, Australia, where molecular analysis was performed. *B. pseudomallei* culture-positive sputum samples were processed as described above. Urine samples included in the analysis were obtained from patients with proven melioidosis who either had a culture-positive urine sample or a non-urine culture-positive site. For example, culture-negative urine samples obtained within 1 day from patients with proven *B. pseudomallei* bloodstream infection were included.

## Data and statistical analysis

The results for the clinical specimen analysis are reported in number and percentage format and include 95% confidence intervals (CIs) where relevant. The MedCalc online diagnostic test evaluation calculator (version 23.2.1) was used to determine sensitivity, positive predictive value (PPV), negative predictive value (NPV) and accuracy. A specificity analysis was not performed in this study, as the molecular target used has been previously extensively evaluated [6]. To assess the agreement between culture and the molecular method, Cohen’s kappa coefficient was used. Interpretation of kappa values includes: ≤0, no agreement; 0.01–0.20, none to slight; 0.21–0.40, fair; 0.41–0.60, moderate; 0.61–0.79, substantial; 0.80–0.9, strong; 0.91–1.00, almost perfect agreement.

## Results

### Analytical sensitivity analysis

**Fig. 1.**
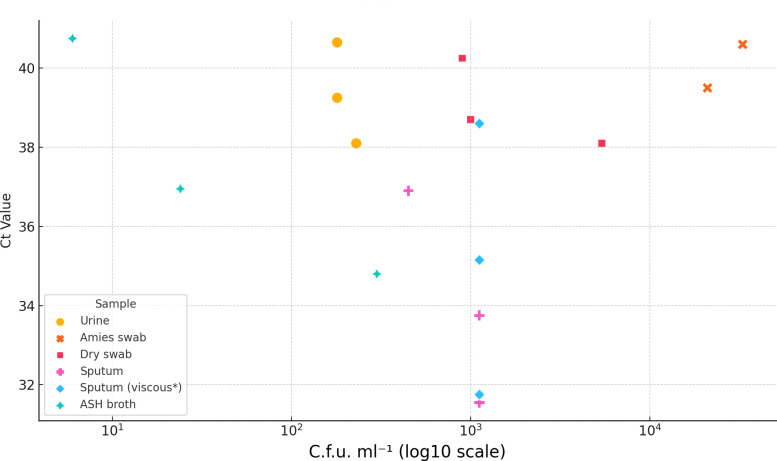
Comparative detection limits by sample type. *Dense, non-liquid mucous.

The Panther^®^ was able to detect *B. pseudomallei* in all sample types included in this analysis. Urine was the clinical sample type with the lowest limit of detection (LOD) (Fig. 1). Eleven samples with an LOD of 1.8×10^2^–7.0×10^2^ c.f.u. ml^−1^ were detected, with 21/22 (95%) replicates positive (Table S1, available in the online Supplementary Material).

An initial evaluation of both dry and Amies swabs revealed an equal absorption volume of ~150 µl of fluid. A concentration of at least 2.1×10^4^ c.f.u. ml^−1^ was required for organism detection when using swabs placed in Amies media, compared with an LOD of 1.0×10^3^ c.f.u. ml^−1^ for detection from dry swabs. The Amies swab was outperformed by the dry swab, which required up to 20,000 less c.f.u. ml^−1^ to detect the organism (Tables S2 and S3).

Compared with other clinical specimens, sputum samples are heterogeneous, ranging from clear, flowing liquid to thick, purulent, tenacious and immobile material. For ease of analysis, sputum samples were divided into viscous (tenacious and immobile) and non-viscous. Initial experiments utilizing our standard laboratory protocol of a 1 : 1 ratio with Sputasol^®^ were unsuccessful (Table S4A); therefore, an adjusted ratio (1 : 2) with improved detection was adopted [9]. This protocol identified an LOD of ~1.0×10^3^ c.f.u. ml^−1^ for both types of sputum; however, this is likely to be lower for non-viscous samples.

The use of ASH broth with an abbreviated incubation period is effective, as evidenced by the ability to detect an initial sample concentration as low as 6 c.f.u. ml^−1^. However, abbreviated incubation of low-concentration swab samples in ASH broth for 4 or 6 h did not improve detection (Tables S5A & B).

As might be predicted, the centrifugation of samples did demonstrate a net benefit for organism detection. Samples taken from the post-centrifuged pellet (bottom of tube) required, on average, 3.4 less cycles to detect the organism (Table 1). This represents an improved diagnostic sensitivity.

**Table 1.** Impact of centrifugation on assay performance

Run	Sample type	c.f.u. ml^-1^	Ct value (mean)	ΔCt (mean)
**Run 1**	Saline (top)	1.5×10⁸	24.0	3.6
	Saline (bottom)	1.5×10⁸	20.4	
**Run 2**	Urine (top)	1.5×10⁶	30.1	4.0
	Urine (bottom)	1.5×10⁶	26.1	
**Run 3**	ASH broth (top)	1.0×10³	38.4	3.2
	ASH broth (bottom)	1.0×10³	35.2	
	ASH broth (top)	9.9×10²	37.5	2.7
	ASH broth (bottom)	9.9×10²	34.8	

## Clinical specimen analysis

Twenty-one culture-positive sputum samples from patients with melioidosis were analysed. The Panther^®^ assay detected *B. pseudomallei* in all cases, including all ten samples with scant growth (Table 2). Two low-volume samples were also successfully detected, with one (Patient 13) showing a late Ct value (Table S6). Comparisons with the contrived dilution series suggest that scant growth corresponds to a bacterial load of ≥10³ c.f.u. ml^−1^.

**Table 2.** Panther^®^ results from clinical samples of patients with melioidosis

Sample type	Culture result	No. of samples	*Burkholderia pseudomallei* PCR detection rate
**Urine**	Culture-negative (no growth)	10	4/10 (40%)
	Mixed flora (non-*Bp*)	4	3/4 (75%)
	*B. pseudomallei* (*Bp* 10^6^–10^8^)	14	14/14 (100%)
**Sputum**	Scant growth *Bp*	10	10/10 (100%)
	1+ growth *Bp*	5	5/5 (100%)
	2+ to 3+ growth *Bp*	6	6/6 (100%)

A total of 28 urine samples from confirmed melioidosis cases were analysed. Among them, 14 were culture-positive for *B. pseudomallei*, all of which were detected by PCR (100%) (Table 2). The remaining 14 were culture-negative, but PCR identified *B. pseudomallei* in 7 (50%) of these cases. In three of these, detection was observed in only one replicate, suggesting a low concentration of organism (Table S7).

Overall, the sensitivity of the molecular assay compared with culture was 100% (CI: 92–100%), with a 100% PPV (CI: 92–100%), 100% NPV (CI: 59–100%) and accuracy of 100% (CI: 93–100%) (Table S8). The kappa coefficient for a culture-positive sample was 1.0.

## Discussion

This study demonstrates the ability of the Panther^®^ molecular diagnostic instrument to detect *B. pseudomallei* directly from various contrived patient samples. The results also reveal the ability of the assay to detect *B. pseudomallei* in culture-positive sputum samples, and in both culture-positive and -negative urine samples from patients with proven melioidosis. This method of culture-independent identification has the ability to improve time to diagnosis.

Genitourinary melioidosis infections occur in 12% of Australian cases [10]. A study of male patients with melioidosis reported that 21% were culture-positive from urine samples. Furthermore, 77% of patients with prostatic abscess were urine culture-positive [11]. Notably, in keeping with international guidelines, many laboratories, including Pathology Queensland, do not routinely report bacterial growth concentrations <10^6^ c.f.u. ml^−1^ [12]. A previous study reported a median concentration of *B. pseudomallei* in urine samples of 1.5×10^4^ c.f.u. ml^−1^ [13]. Considering the LOD reported in this study and the limitations of urine culture, direct molecular detection may not only improve time to diagnosis of genitourinary and prostate infection, but it is also likely to improve the diagnostic sensitivity of both clinical syndromes.

As pneumonia is the most common clinical manifestation of melioidosis, the ability to identify the pathogen directly from a sputum sample would be beneficial [1]. Performing PCR directly on sputum has been shown to improve diagnostic sensitivity [14]. The median burden of organism in sputum of 1.1×10^5^ c.f.u. ml^−1^ is considerably higher than that required for identification with the Panther^®^ [13]. The real-world sputum results of this study reveal that patients with culture-positive sputum showing scant growth will have a detectable molecular result, with a median Ct value of 33. Based on the contrived experiment results in Table S4B, this likely equates to 10^3–4^ c.f.u. ml^−1^.

Wound swabs placed in Amies media may be used for detection of *B. pseudomallei*; however, this study demonstrated the superiority of dry swabs. This may be due to loss of organism into the media or decreased desorption into the lysis buffer during sample processing. As ASH broth is a common selective medium used for enrichment of *B. pseudomallei*, it is helpful to note that there was no assay inhibition of detection due to the presence of this broth. Disappointingly, the abbreviated incubation of low organism concentration from dry swabs in ASH broth did not improve organism detection. This may be due to the combination of low absorption volume and incomplete desorption. Nevertheless, it is notable that a sample concentration of <10 c.f.u. ml^−1^ can be enriched and detected with a 4 h abbreviated incubation. These results suggest a potential pathway for early detection of other laboratory diagnoses such as bacteraemia, by directly inoculating patient’s blood into ASH broth at presentation.

There are a number of challenges and limitations identified in this study. The LOD, and therefore likely analytical sensitivity, varies depending on sample type. It is important to understand the complex nature of clinical specimens when assessing direct-from-sample diagnostic assays. While urine was reported to have the lowest LOD, the contrived clinical samples used were not evaluated for urea concentration. This is potentially significant, as urea may be an inhibitor of the PCR reaction [15]. The urine samples from proven patients with melioidosis were processed using a standard 1 µl loop plated onto HBA/MacConkey agar and did not undergo centrifugation as compared to the 500 µl urine specimens that were centrifuged before PCR. While this may have improved the diagnostic yield of culture in this patient cohort, all were culture-positive at alternate sites. The aim of this study was not to assess the method of urine sample processing for culture, but rather to evaluate the ability of the Panther^®^ to detect *B. pseudomallei* in real patient samples.

Assessment of swabs for assay performance is challenging. Due to their absorptive nature and ability to saturate, used clinical swabs could not be evaluated. A major limitation to experimenting with unused swabs is the inability to truly assess the capability of the assay to perform within a complex, purulent wound matrix. Additionally, while the swabs’ fluid absorption is relatively consistent, it is difficult to determine the total concentration of organism both absorbed and subsequently desorbed [16].

The results from this study clearly demonstrate a limitation of the Panther^®^ when processing sputum samples. Viscous samples either result in an invalid result (potentially due to a limitation of instrument pipetting) or require an alternative pre-processing method. Even with improved homogenization via an increased ratio of liquefying agent, the results reveal a decreased assay efficiency, as evidenced by the LOD and internal control. However, using proven culture-positive patient sputum, in which the LOD appears sufficient, the assay was able to detect the pathogen in all samples.

This assay used a cycle threshold (Ct) cut-off of ≤43, which exceeds the more commonly applied threshold of ≤40. Additionally, samples with detection in only one of two replicates were classified as positive. While these criteria may increase the potential for false-positive results, interpretation was supported by valid internal controls, both positive and no-template controls included in each run, and consideration of the clinical context, including pre-test probability.

Using culture as the gold standard comparator, the assay used in this study demonstrated an excellent diagnostic accuracy and PPV. It is important to consider the wide CI associated with the NPV. The evaluation of this assay was limited to 49 clinical samples, and a specificity analysis was not explicitly performed; therefore, these results should be interpreted with caution. A negative result may not be sufficient to exclude the diagnosis.

## Conclusion

In summary, the Panther^®^, an automated molecular diagnostic instrument, is able to detect *B. pseudomallei* directly from heterogeneous clinical sample types. The LOD described in this study is lower than previously reported median organism concentrations per sample type. The assay was able to detect the pathogen in all real-world culture-positive clinical samples and is therefore a promising method for further evaluation in the diagnostic laboratory.

## Supplementary material

10.1099/jmm.0.002074 Uncited Supplementary Material 1.

## Abbreviations

ASH: Ashdown’s
CI: confidence interval
Ct: cycle threshold
HBA: horse blood agar
LOD: limit of detection
NPV: negative predictive value
PPV: positive predictive value
SAT: specimen aliquot tube
