# Supplementary material for: Evaluation of an automated molecular diagnostic instrument for direct detection of Burkholderia pseudomallei from clinical specimens
Source: J Med Microbiol. 2025 Sep 22;74(9):002074. doi: 10.1099/jmm.0.002074 (PMC12476149; doi:10.1099/jmm.0.002074)
Supplement: Uncited Supplementary Material 1. [file jmm-74-02074-s001.pdf]

Supplementary Table 1. Results of urine experiments

CFU/ml	Ct values	
	Replicate 1	Replicate 2
Run 1		
3.75 x 10^4	31.2	32.0
1.8 x 10^3	36.4	36.2
2.3 x 10^2	38.5	37.7
2.5 x 10^1	ND	ND
Run 2		
4.5 x 10^4	31.7	31.5
2.2 x 10^3	37.0	37.2
2.8 x 10^2	36.8	38.9
5.3 x 10^1	37.9	39.4
3.2 x 10^1	ND	ND
Run 3		
7.0 x 10^2	36.0	35.8
7.0 x 10^2	38.0	36.1
7.0 x 10^2	38.1	ND
4.5 x 10^2	38.5	36.6
4.5 x 10^2	36.8	37.9
4.0 x 10^2	40.8	37.0
Run 4		
2.6 x 10^2	ND	39.5
1.8 x 10^2	39.7	38.8
1.8 x 10^2	41.9	39.4
1.4 x 10^2	ND	ND
1.0 x 10^2	ND	ND
Run 5		
4.9 x 10^1	39.9	ND
3.3 x 10^1	ND	ND
2.4 x 10^1	ND	ND
1.6 x 10^1	ND	ND
0.7 x 10^1	ND	ND
	Internal control range: 28.6 – 30.4	

Supplementary Table 2. Results of Amies swab experiments

CFU/ml	Ct values	
	Replicate 1	Replicate 2
Run 1		
3.3 x 10^4	40.2	41.0
6.4 x 10^3	ND	ND
Run 2		
2.1 x 10^4	ND	39.5
5.4 x 10^3	ND	ND
Run 3		
6.6 x 10^2	ND	ND
6.6 x 10^2	ND	ND
6.6 x 10^2	ND	ND
	Internal control range: 28.5 – 28.8	

Supplementary Table 3. Results of dry swab experiments

CFU/ml	Ct values	
	Replicate 1	Replicate 2
Run 1		
3.3 x 10^4	33.9	33.9
6.4 x 10^3	35.5	36.4
9.4 x 10^2	41.6	ND
Run 2		
2.1 x 10^4	35.1	35.0
5.4 x 10^3	37.8	38.4
Run 3		
8.7 x 10^3	37.7	39.4
8.5 x 10^3	36.8	37.3
8.0 x 10^3	36.3	38.3
Run 4		
1.0 x 10^3	38.7	ND
9.6 x 10^2	38.4	ND
9.0 x 10^2	38.3	42.2
	Internal control range: 29.0 – 30.3	

Supplementary Table 4 A. Results of sputum experiments using 1:1 Sputasol®

CFU/ml	Ct values	
	Replicate 1	Replicate 2
Run 1 (Non-viscous)		
6.8x10^4	31.3	30.4
6.6x10^4	34.4	34.3
1.7x10^4	33.2	32.0
1.6x10^4	31.8	32.1
	Internal control range 30.4 – 43.2	
Run 2 (Viscous)		
3.1x10^4	Failed	Failed
1.7x10^4	Failed	Failed
1.7x10^4	Failed	Failed
	Internal control range: Failed	
Run 3 (Non-viscous)		
3.5x10^2	39.3	ND
3.5x10^2	ND	ND
3.5x10^2	ND	ND
	Internal control range: 33.1 – 36.6	
Run 4 (Non-viscous)		
7.5x10^1	ND	ND
7.5x10^1	ND	ND
4.0x10^1	ND	ND
4.0x10^1	ND	ND
4.0x10^1	ND	ND
	Internal control range: 29.3 – 35.4	

Supplementary Table 4 B. Results of sputum experiments using 1:2 Sputasol®

CFU/ml	Ct values	
	Replicate 1	Replicate 2
Run 1 (Non-viscous)		
1.12 x 10^3	33.8	33.7
1.12 x 10^3	31.1	32.0
1.12 x 10^3	35.4	34.6
	Internal control range 29.6 – 36.1	
Run 2 (Viscous)		
1.12 x 10^3	36.9	33.4
1.12 x 10^3	31.5	32.0
1.12 x 10^3	36.8	40.4
	Internal control range 31.1 – 37.1	
Run 3 (Non-viscous)		
4.5 x 10^2	37.6	36.2
4.8 x 10^2	39.1	40.0
5.0 x 10^2*	ND	INV
	Internal control range 36.0 – 38.6	

Supplementary Table 5 A. Results of Ashdown's broth experiments

CFU/ml pre-incubation	Ct values	
	Replicate 1	Replicate 2
Run 1: 4-hour broth incubation		
3.0 x 10^2	34.4	35.2
2.4 x 10^1	35.9	38.0
0.6 x 10^1	40.3	41.2
Run 2: 6-hour broth incubation		
3.0 x 10^2	32.0	33.0
2.4 x 10^1	36.7	37.5
0.6 x 10^1	42.7	38.2
	Internal control range: 29.3 – 30.1	

Supplementary Table 5 B. Results of dry swab into Ashdown's broth experiments

CFU/ml pre-incubation*	Ct values	
	Replicate 1	Replicate 2
Run 1: 4-hour broth incubation		
1.8 x 10^2	ND	ND
1.4 x 10^1	ND	ND
0.3 x 10^1	ND	ND
Run 2: 6-hour broth incubation		
1.8 x 10^2	38.6	37.4
1.4 x 10^1	ND	ND
0.3 x 10^1	ND	ND
	Internal control range: 29.7 – 30.3	

*Accounting for a swab volume absorption of 150ul

Supplementary Table 6. Panther® results from clinical sputum samples of melioidosis patients

Sample	Lab report	CT values	
		Replicate 1	Replicate 2
Patient 1	Scant	31.4	31.3
Patient 2	Scant	32.7	32.5
Patient 3	Scant	36.8	36.5
Patient 4	Scant	35.0	35.7
Patient 5	Scant	33.9	34.0
Patient 6	Scant	32.2	32.0
Patient 7	Scant	30.5	31.0
Patient 8	Scant	34.9	35.1
Patient 9	Scant	32.4	33.3
Patient 10	Scant	30.3	29.9
Patient 11	1+	30.3	31.0
Patient 12	1+	29.7	29.5
Patient 13*	1+	35.9	37.0
Patient 14	1+	30.0	30.1
Patient 15	1+	26.4	26.9
Patient 16	2+	30.7	30.7
Patient 17	2+	27.6	26.9
Patient 18	2+	31.0	31.2
Patient 19	2+	26.4	26.3
Patient 20	3+	29.2	29.6
Patient 21*	3+	30.9	31.1

Sputum results are routinely reported as: no growth; 1+, 2+; 3+ to denote relative growth; *denotes low volume sputum ≤0.5ml

Supplementary Table 7. Panther® results from clinical urine samples of melioidosis patients

Sample	Laboratory report	Replicate 1	Replicate 2
Patient 1	No growth	40.8	ND
Patient 2	No growth	ND	38.9
Patient 3	No growth	ND	ND
Patient 4	No growth	42.9	40.9
Patient 5	No growth	36.8	ND
Patient 6	No growth	ND	ND
Patient 7	No growth	ND	ND
Patient 8	No growth	ND	ND
Patient 9	No growth	ND	ND
Patient 10	No growth	ND	ND
Patient 11	10^6 mixed skin flora only	36.3	36.4
Patient 12	10^7 mixed enterics	ND	ND
Patient 13	10^7 mixed skin flora only	32.2	32.0
Patient 14	10^8 Candida sp. only	36.0	35.3
Patient 15	Bp 10^6	29.3	29.7
Patient 16	Bp 10^7	30.0	29.5
Patient 17	Bp 10^7	27.6	27.7
Patient 18	Bp 10^7	32.7	34.0
Patient 19	Bp 10^7	27.4	27.6
Patient 20	Bp 10^8	28.1	28.2
Patient 21	Bp 10^8	22.2	21.3
Patient 22	Bp 10^8	25.3	26.0
Patient 23	Bp 10^8	21.2	20.8
Patient 24	Bp 10^8	28.6	28.7
Patient 25	Bp 10^8	26.6	26.7
Patient 26	Bp 10^8	24.0	23.9
Patient 27	Bp 10^8	24.9	25.3
Patient 28	Bp 10^8 (+10^7 mixed skin flora)	19.5	19.8

Supplementary Table 8. Confusion matrix comparing culture to PCR (reference standard)

A. Urine clinical samples

Culture		Disease		
		**Present**	**Absent**	
Test	Positive	14	0	PPV: 100% (77-100)
	Negative	7	7	NPV: 99% (99-100)
	Sens/Spec: % (range)	67% (43-85)	100% (59-100)	
**PCR**		**Present**	**Absent**	
Test	Positive	21	0	PPV: 100% (84-100)
	Negative	0	7	NPV: 99% (59-100)
	Sens/Spec: % (range)	100% (84-100)	100% (59-100)	

B. All clinical samples

PCR		Disease		
		**Present**	**Absent**	
Test	Positive	42	0	PPV: 100% (92-100)
	Negative	0	7	NPV: 99% (59-100)
	Sens/Spec: % (range)	100% (92-100)	100% (59-100)	

Assuming a 1% prevalence and disease identified as a PCR positive result
